# Supplementary material for: The origins of the Guinness stout yeast
Source: Commun Biol. 2024 Jan 12;7:68. doi: 10.1038/s42003-023-05587-3 (PMC10786833; doi:10.1038/s42003-023-05587-3)
Supplement: Supplementary file 2 — Supplementary Information [file 42003_2023_5587_MOESM2_ESM.pdf]

The Origins of the Guinness Stout Yeast

Daniel W. M Kerruish[1], Paul Cormican[2], Elaine M. Kenny[2], Jessica Kearns[1], Eibhlin Colgan[1], Chris A. Boulton[3] and

Sandra N.E Stelma[1].

[1]Diageo Ireland, St James's Gate, The Liberties, Dublin, Ireland

[2]ELDA biotech, Kildare, Ireland

[3]Brewing Consultant, Burton on Trent, UK

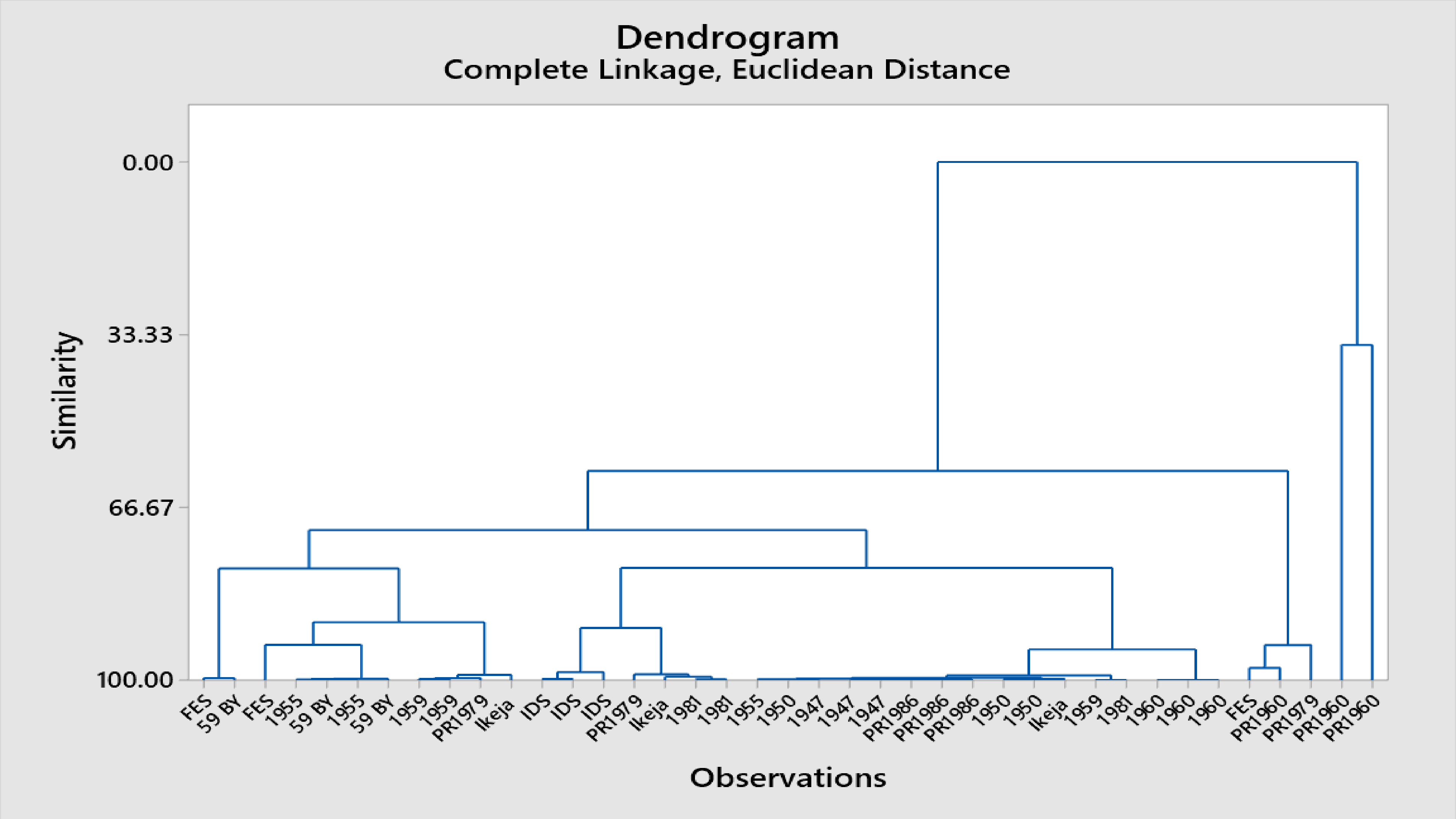

Supplementary Figure 1: Hierarchical clustering analysis of PCR products (determined using Bioanaylzer 2.0 DNA chips) of the interdelta specific primers δ2/ δ 12[1] PCRs were conducted on typical and atypical Guinness yeast morphology as observed through Giant Colony Morphology.

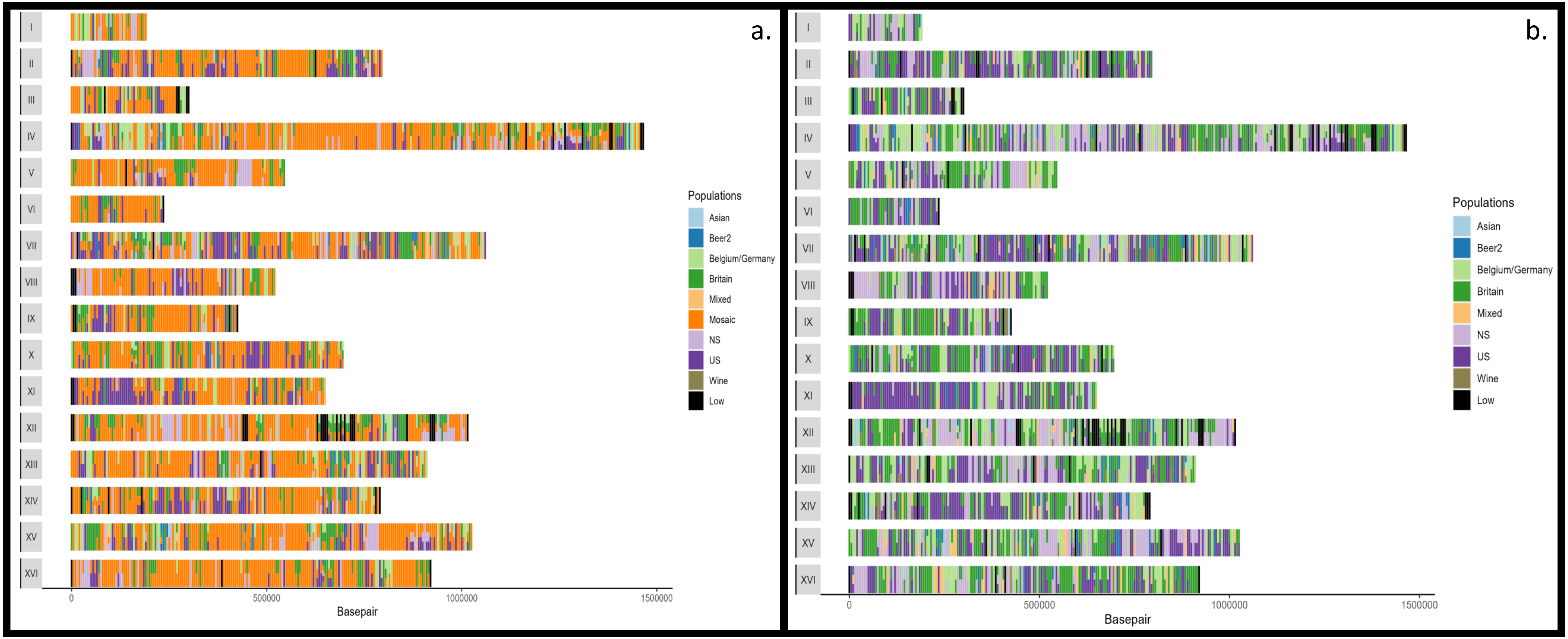

Supplementary Figure 2: Chromosome admixture of the representative Guinness yeast strain IDS1 using the sequence data set of *S. cerevisiae* (BioProject PRJNA323691)[2]. Admixture of IDS1 was determined using Alpaca (v1)[3] software with a kmer length of 21 over 5000 base pair sliding windows. Plot data in a. and b. include and omit the mosaic yeast Beer042.

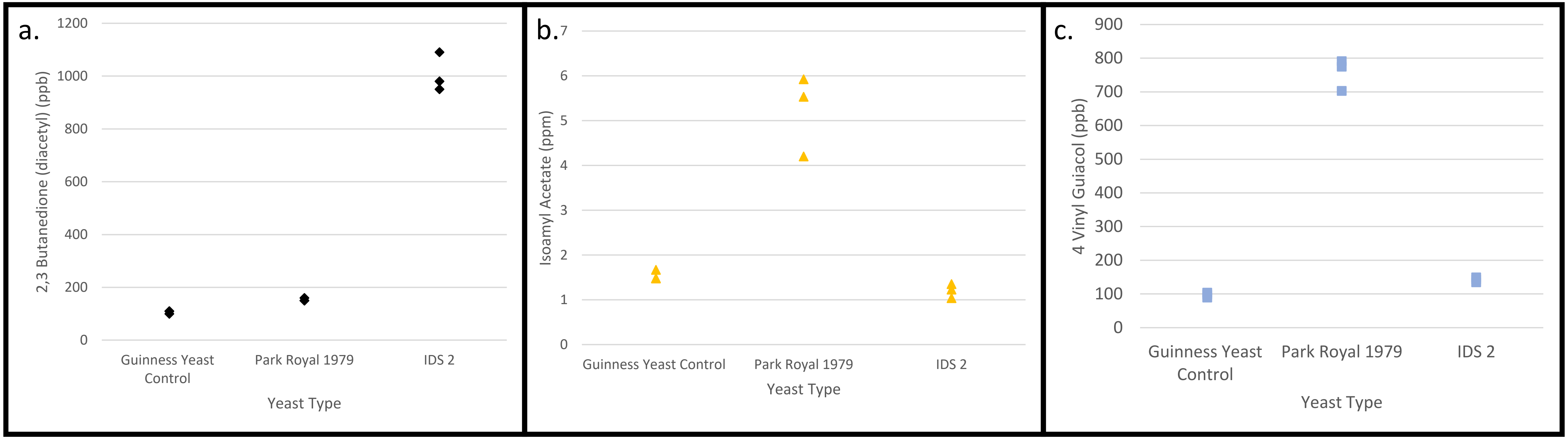

Supplementary Figure 3: Concentration of (a.) 2, 3 Butanedione (diacetyl), (b.) Isoamyl Acetate and (c.) 4 Vinyl Guaiacol of Guinness Irish Draught Stout brewed using a control Guinness yeast from Dublin St James's Gate and the Guinness yeasts, Park Royal 1979 and IDS 2. All fermentations were conducted in 100L fermentation vessels with Guinness wort collected from St James's Gate Brewery. Observations presented are n=3 biologically independent samples.

Software	Reference	Source
trimgalore (Version 0.6.1)	Babraham Bioinformatics	https://github.com/FelixKrueger/TrimGalore
BWA (Version 0.7.17).	Li and Durbin 2009 (4)	http://bio-bwa.sourceforge.net/
SamTools	Daneck *et al.,* 2021 (5)	https://github.com/samtools/samtools
Ragtag (version 1.0.2)	Alonge et al., 2019 (6)	https://github.com/malonge/RagTag
Picard (Version 2.18.23).	Broad Institute	https://github.com/broadinstitute/picard
qualimap (Version 2.2.1	Okonechnikov et al 2016 (7)	http://qualimap.conesalab.org/doc_html/intro.html
SPAdes (Version 3.14	Bankevich et al., 2012 (8)	https://cab.spbu.ru/software/spades/
Guppy (version 3.6)	Oxford Nanopore Technologies Ltd	https://community.nanoporetech.com/protocols/Guppy-protocol/v/gpb_2003_v1_revl_14dec2018/linux-guppy
Nanofilt	De Coster et al., 2018 (9)	https://github.com/wdecoster/nanofilt
Porechop (version 6)	Murigneux et al., 2020 (10)	https://github.com/rrwick/Porechop
Seqtk (version 1.3)	MIT Licence	https://github.com/lh3/seqtk
Flye (version 2.8)	Kolmogorov et al., 2020 (11)	https://github.com/fenderglass/Flye
Medaka (version 1.0.3)	Oxford Nanopore Technologies Ltd	https://github.com/nanoporetech/medaka
Quast 5.10.0	Qusat	https://github.com/ablab/quast
Busco (version 3)	Waterhouse et al., 2018 (12)	https://gitlab.com/ezlab/busco
Funannotate (version 1.74)	Palmer and Stajich 2019 (13)	https://github.com/nextgenusfs/funannotate
GATK (version 4.1.4-1)	Van der Auwera and O'Connor 2020 (14)	https://github.com/broadinstitute/gatk/releases
SnpEff (version 4.3)	Cingolani et al., 2012 (15)	http://pcingola.github.io/SnpEff/
Orthofinder (version 2.3.3)	Emms and Kelly 2019 (16)	https://github.com/davidemms/OrthoFinder
MUSCLE (version 3.8.31)	European Bioinformatics Institute	https://www.ebi.ac.uk/Tools/msa/muscle/
RAxML (version 8)	Stamatakis 2014 (17)	https://github.com/stamatak/standard-RAxML
ggtree (version 3.6.2)	G Yu et al., 2017 (18)	https://yulab-smu.top/
Plink (version 1.09)	Purcell et al., 2007 (19)	https://www.cog-genomics.org/plink/
fastSTRUCTURE (version 10)	Raj et al., 2014 (20)	https://rajanil.github.io/fastStructure/
Alpaca (version 1)	Salazar and Abeel 2019 (3)	https://github.com/AbeelLab/Alpaca
Control -FREEC (version 5.7)	Boeva *et al.,* 2012 (21)	https://github.com/BoevaLab/FREEC
Fijarczyk2020_JeanTalon	Fijarczyk *et al.,* 2020 (22)	https://github.com/Landrylab/Fijarczyk2020_JeanTalon

Supplementary Table 1: Bioinformatic programmes used in this study.

References:

1. Legras, J.L. and Karst, F. Optimisation of interdelta analysis for Saccharomyces cerevisiae strain characterisation. *FEMS microbiology letters*, *221*(2), pp.249-255 (2003).

2. Gallone, B., Steensels, J., Prahl, T., Soriaga, L., Saels, V., Herrera-Malaver, B., Merlevede, A., Roncoroni, M., Voordeckers, K., Miraglia, L. and Teiling, C. Domestication and divergence of Saccharomyces cerevisiae beer yeasts. *Cell*, *166*(6), pp.1397-1410 (2016).

3. Salazar, A.N. and Abeel, T., 2019. Alpaca: a kmer-based approach for investigating mosaic structures in microbial genomes. *bioRxiv*, p.551234

4. Li, H. and Durbin, R. Fast and accurate short read alignment with Burrows–Wheeler transform. *bioinformatics*, *25*(14), pp.1754-1760 (2009).

5. Danecek, P., Bonfield, J.K., Liddle, J., Marshall, J., Ohan, V., Pollard, M.O., Whitwham, A., Keane, T., McCarthy, S.A., Davies, R.M. and Li, H., 2021. Twelve years of SAMtools and BCFtools. *Gigascience*, *10*(2), p.giab008.

6. Alonge, M., Soyk, S., Ramakrishnan, S., Wang, X., Goodwin, S., Sedlazeck, F.J., Lippman, Z.B. and Schatz, M.C. RaGOO: fast and accurate reference-guided scaffolding of draft genomes. *Genome biology*, *20*(1), pp.1-17 (2019).

7. Okonechnikov, K., Conesa, A. and García-Alcalde, F. Qualimap 2: advanced multi-sample quality control for high-throughput sequencing data. *Bioinformatics*, *32*(2), pp.292-294 (2016).

8. Bankevich, A., Nurk, S., Antipov, D., Gurevich, A.A., Dvorkin, M., Kulikov, A.S., Lesin, V.M., Nikolenko, S.I., Pham, S., Prjibelski, A.D. and Pyshkin, A.V. SPAdes: a new genome assembly algorithm and its applications to single-cell sequencing. *Journal of computational biology*, *19*(5), pp.455-477 (2012).

9. De Coster, W., D'Hert, S., Schultz, D.T., Cruts, M. and Van Broeckhoven, C. NanoPack:visualizing and processing long-read sequencing data. *Bioinformatics*, *34*(15), pp.2666-2669 (2018).

10. Murigneux, V., Rai, S.K., Furtado, A., Bruxner, T.J., Tian, W., Harliwong, I., Wei, H., Yang, B., Ye, Q., Anderson, E. and Mao, Q. Comparison of long-read methods for sequencing and assembly of a plant genome. *GigaScience*, *9*(12), p.giaa146 (2020).

11. Kolmogorov, M., Bickhart, D.M., Behsaz, B., Gurevich, A., Rayko, M., Shin, S.B., Kuhn, K., Yuan, J., Polevikov, E., Smith, T.P. and Pevzner, P.A. metaFlye: scalable long-read metagenome assembly using repeat graphs. *Nature Methods*, *17*(11), pp.1103-1110 (2020).

12. Waterhouse, R.M., Seppey, M., Simão, F.A., Manni, M., Ioannidis, P., Klioutchnikov, G., Kriventseva, E.V. and Zdobnov, E.M. BUSCO applications from quality assessments to gene prediction and phylogenomics. *Molecular biology and evolution*, *35*(3), pp.543-548 (2018).

13. Palmer, J. & Stajich, J. nextgenusfs/funannotate: funannotate v1.5.3 (Version 1.5.3). Zenodo. https://doi.org/10.5281/zenodo.2604804 (2019)

14. Van der Auwera, G.A. and O'Connor, B.D. *Genomics in the cloud: using Docker, GATK, and WDL in Terra*. O'Reilly Media (2020).

15. Cingolani, P., Platts, A., Wang, L.L., Coon, M., Nguyen, T., Wang, L., Land, S.J., Lu, X. and Ruden, D.M. A program for annotating and predicting the effects of single nucleotide polymorphisms, SnpEff: SNPs in the genome of Drosophila melanogaster strain w1118; iso-2; iso-3. *Fly*, *6*(2), pp.80-92 (2012).

16. Emms, D.M. and Kelly, S., 2019. OrthoFinder: phylogenetic orthology inference for comparative genomics. *Genome biology*, *20*, pp.1-14.

17. Stamatakis, A., 2014. RAxML version 8: a tool for phylogenetic analysis and post-analysis of large phylogenies. *Bioinformatics*, *30*(9), pp.1312-1313.

18. Yu, G., Lam, T.T.Y., Zhu, H. and Guan, Y., 2018. Two methods for mapping and visualizing associated data on phylogeny using ggtree. *Molecular biology and evolution*, *35*(12), pp.3041-3043.

19. Purcell, S., Neale, B., Todd-Brown, K., Thomas, L., Ferreira, M.A., Bender, D., Maller, J., Sklar, P., De Bakker, P.I., Daly, M.J. and Sham, P.C., 2007. PLINK: a tool set for whole-genome association and population-based linkage analyses. *The American journal of human genetics*, *81*(3), pp.559-575.

20. Raj, A., Stephens, M. and Pritchard, J.K., 2014. fastSTRUCTURE: variational inference of population structure in large SNP data sets. *Genetics*, *197*(2), pp.573-589.Salazar, A.N. and Abeel, T., 2019. Alpaca: a kmer-based approach for investigating mosaic structures in microbial genomes. *bioRxiv*, p.551234.

21. Boeva, V., Popova, T., Bleakley, K., Chiche, P., Cappo, J., Schleiermacher, G., Janoueix-Lerosey, I., Delattre, O. and Barillot, E., 2012. Control-FREEC: a tool for assessing copy number and allelic content using next-generation sequencing data. *Bioinformatics*, *28*(3), pp.423-425.

22. Fijarczyk, A., Hénault, M., Marsit, S., Charron, G., Fischborn, T., Nicole-Labrie, L. and Landry, C.R., 2020. The genome sequence of the Jean-Talon strain, an archaeological beer yeast from Québec, reveals traces of adaptation to specific brewing conditions. *G3: Genes, Genomes, Genetics*, *10*(9), pp.3087-3097.
