# Supplementary material for: The origins of the Guinness stout yeast
Source: Commun Biol. 2024 Jan 12;7:68. doi: 10.1038/s42003-023-05587-3 (PMC10786833; doi:10.1038/s42003-023-05587-3)
Supplement: Supplementary file 3 — Description of Additional Supplementary Files [file 42003_2023_5587_MOESM3_ESM.docx]

**Description of Additional Supplementary Files**

**File name:** Supplementary Data 1

**Description:** SNPs of MAL11 and MAL31 genes

**File name:** Supplementary Data 2

**Description:** CNV of the genes responsible for the Ehrlich pathway

**File name:** Supplementary Data 3

**Description:** SNPs of ILV2 and ILV6 genes

**File name:** Supplementary Data 4

**Description:** SNPs of FLO genes

**File name:** Supplementary Data 5

**Description:** The origins of the Guinness yeast raw data files
